# Treatment and risk of relapse in GCA in Western Norway 2013–2020: a retrospective cohort study

**DOI:** 10.1093/rap/rkaf109

**Published:** 2025-09-20

**Authors:** Hans K Skaug, Bjørg-Tilde S Fevang, Jörg Aßmus, Andreas P Diamantopoulos, Geirmund Myklebust, Lene K Brekke

**Affiliations:** Department of Rheumatology, Haugesund Hospital for Rheumatic Diseases, Haugesund, Norway; Department of Clinical Science (K2), Faculty of Medicine, University of Bergen, Bergen, Norway; Bergen Group of Epidemiology and Biomarkers in Rheumatic Disease (BEaBIRD), Department of Rheumatology, Haukeland University Hospital, Bergen, Norway; Department of Clinical Science (K2), Faculty of Medicine, University of Bergen, Bergen, Norway; Bergen Group of Epidemiology and Biomarkers in Rheumatic Disease (BEaBIRD), Department of Rheumatology, Haukeland University Hospital, Bergen, Norway; Centre for Clinical Research, Haukeland University Hospital, Bergen, Norway; Division of Internal Medicine, Department of Infectious Diseases, Akershus University Hospital, Nordbyhagen, Norway; Research Department, Hospital of Southern Norway, Kristiansand, Norway; Department of Rheumatology, Haugesund Hospital for Rheumatic Diseases, Haugesund, Norway; Bergen Group of Epidemiology and Biomarkers in Rheumatic Disease (BEaBIRD), Department of Rheumatology, Haukeland University Hospital, Bergen, Norway

**Keywords:** large vessel vasculitis, GCA, treatment, relapse

## Abstract

**Objectives:**

During recent decades, there has been a growing recognition of the spectrum of GCA. This study aims to identify predictors of variation in the initial treatment of GCA and compare the relapse risk among patients with four different clinical phenotypes using a cohort of 256 well-defined GCA patients in Western Norway.

**Methods:**

Regression models were used to identify predictors of differences in initial oral glucocorticoid (GC) dosage and for the administration of intravenous GC (IVGC). Tapering of GCs was analysed using a linear mixed effects model. Time to GC discontinuation, end of rheumatological follow-up, and relapse were assessed with Kaplan–Meier methods and Cox regression.

**Results:**

Patients with cranial phenotype had lower risk of relapse, were more likely to discontinue GC treatment, and had shorter follow-up time at the rheumatological department compared with patients with other phenotypes. Neither the initial GC treatment nor GC tapering differed between the phenotypes. The only factor strongly associated with more intensive initial treatment was visual disturbances.

**Conclusions:**

Patients with the cranial GCA phenotype seem to have a shorter and less complicated disease course compared with patients with other phenotypes. This could suggest that early phenotype identification can yield important prognostic information.

## Graphical Abstract

**Figure:**
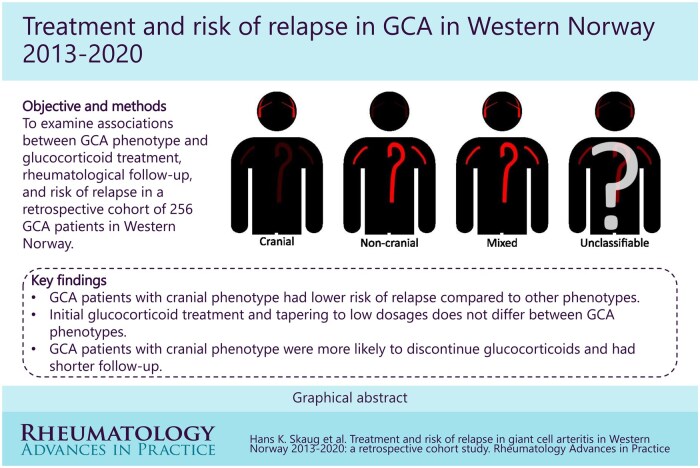

Key messages

- GCA patients with cranial phenotype had a lower risk of relapse compared to other phenotypes.
- Initial glucocorticoid (GC) treatment and tapering to low dosage do not differ between GCA phenotypes.
- GCA patients with cranial phenotype were more likely to discontinue GCs and had shorter follow-up.

## Introduction

GCA is a large vessel vasculitis affecting people aged 50 years and older. In recent decades, there has been an increasing interest in the different phenotypes of GCA, that is, the distribution of arterial wall inflammation [1]. A distinction is made between ‘classical’ cranial GCA and GCA with involvement of non-cranial (large) arteries, the latter phenotype often referred to as large vessel GCA (LV-GCA). The large vessels referred to are mainly the aorta and the axillary, carotid, subclavian, and vertebral arteries. The clinical presentation of GCA is heterogeneous, and some features overlap between the different phenotypes [2, 3]. Thus, to fully assess GCA with regard to phenotype, it is now common practice to use imaging diagnostics. Recent recommendations for management of GCA reflect this, with vascular ultrasound emerging as a modality of choice [4–6].

High-dosage GC therapy is the mainstay of GCA treatment, and alternative treatment options are limited. Among the steroid-sparing DMARDs in use, tocilizumab has the best documented effect [7–10]. Other steroid-sparing options include methotrexate and leflunomide, although the supporting evidence is limited [11–14]. Still, no medical therapy has been shown to be able to eliminate the need for GC.

A lack of consensus on the definition of relapse has limited the comparability of earlier studies addressing the prevalence of relapse in GCA populations [15]. However, recent guidelines have presented definitions of what constitutes a GCA relapse [5, 16]. Studies addressing risk factors for relapse show conflicting results, but LV-GCA has repeatedly been shown to be associated with a higher risk of relapse [17–24]. Still, the implications of this knowledge in the early management of GCA remain unclear.

In this study, we aim to gain an understanding of how different factors influence treatment and disease course in GCA patients. The objective is to address differences in treatment and rheumatological follow-up and compare the relapse risk between four clinical phenotypes using our unique cohort of 256 well-defined GCA patients in Western Norway.

## Methods

The study population comprises 256 GCA patients diagnosed in the Bergen Health Area from 1 January 2013 to 31 December 2020. Data were collected by retrospective investigation of electronic patient records. The inclusion process and patient characteristics have been described in a previous publication [3]. Data from the inclusion period and a further 2.25 years of follow-up were collected according to a standardized data collection form [3]. Thus, the total study period is from 1 January 2013 to 31 March 2023. Data were registered for as long as the patients remained under follow-up by the rheumatology department during the study period.

**Figure 1.**
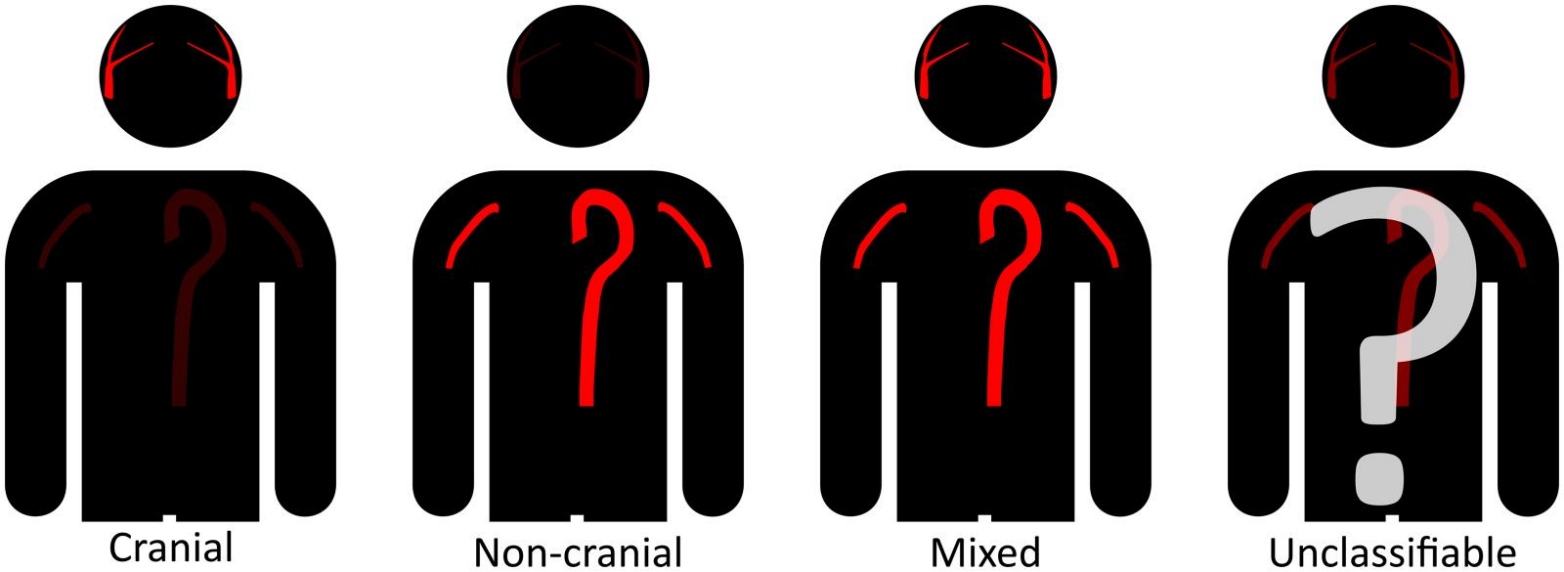
Phenotype definitions. Phenotype definitions based on biopsy and imaging results

Symptoms and clinical findings were registered as present or absent, and any sign or symptom was assumed to be absent if not described in the patient records. Patients were only included in the cohort if they were deemed clinically similar to GCA cases, and if they fulfilled at least one out of three sets of classification criteria. We defined four phenotypes of GCA based on the results of temporal artery biopsy (TAB) and imaging diagnostics (Fig. 1). Patients with a positive TAB or a positive vascular ultrasound of the cranial arteries, without documented large vessel involvement, were classified as having cranial phenotype. Patients were classified as having non-cranial phenotype if they had large vessel involvement documented by vascular ultrasound of the axillary arteries, PET/CT, magnetic resonance angiography, or CT angiography, without documented involvement of cranial arteries. Patients overlapping the two categories were classified as having a mixed phenotype. Patients without positive findings from examinations of the cranial arteries and examinations of the non-cranial arteries were classified as unclassifiable phenotype. The prednisone equivalent dosage (mg/day) of GC treatment was collected at the time of treatment initiation and after 3, 6, 12, and 24 months. We defined active disease, remission, and relapse in accordance with the EULAR recommendations [16]. Active disease was defined as the presence of typical clinical symptoms or signs of active vasculitis and either persistently elevated inflammatory markers (with other possible causes ruled out), signs of active vasculitis on imaging, or ischaemic complications attributable to vasculitis. Remission was defined as the absence of all clinical signs and symptoms attributable to active disease. Relapse was defined as recurrence of active disease in a patient in remission, with a subsequent treatment intensification equivalent to an increased prednisone dosage of at least 5 mg/day. We classified relapses as major or minor [16]. Patients who did not experience a relapse were included in the analysis of GC tapering. GC treatment was recorded as successfully discontinued, given documented discontinuation without any records of reinitiation for at least 6 months.

### Statistical analyses

Descriptive statistics are presented as mean with S.D. or median with interquartile range (IQR). Categorical variables are presented as counts with percentages (Table 1). Comparisons of categorical variables were performed using the chi-squared test or Fisher’s test as appropriate.

**Table 1.** Population characteristics

	Phenotype				
Patient characteristic	Cranial (*N* = 159)	Non-cranial (*N* = 39)	Mixed (*N* = 20)	Unclassifiable (*N* = 38)	Total (*N* = 256)
Sex, *n* (%)					
Female	108 (68)	28 (72)	13 (65)	31 (82)	180 (70)
Male	51 (32)	11 (28)	7 (35)	7 (18)	76 (30)
Age at diagnosis (years)^a^	74 (69–81)	66 (58–74)	67 (65–74)	70 (63–80)	72 (66–79)
Diagnostic delay (days)^a^	39 (19–89)	80 (59–149)	86 (62–130)	35 (15–64)	51 (22–95)
Positive temporal artery biopsy, *n* (%)	152 (98%)	0	14 (82%)	0	166 (71%)
Large vessel involvement, *n* (%)	0	39 (100)	20 (100)	0	59 (23)
Serum markers					
CRP (mg/L)^a^	71 (40–115)	80 (60–117)	62 (42–120)	54 (33–85)	69 (41–114)
ESR (mm/1 h)^a^	79 (57–96)	104 (73–111)	91 (72–105)	66 (40–98)	80 (56–101)
Haemoglobin (g/dl)^a^	12.30 (11.35–13.20)	11.20 (10.30–12.80)	10.75 (10.20–12.60)	12.55 (11.40–13.20)	12.10 (11.00–13.10)
Platelet count (×10^9^/L)^a^	405 (328–472)	429 (322–518)	420 (343–488)	342 (279–461)	405 (317–475)
Treatment					
GC dose at treatment initiation (mg)^a^	50 (40–60)	50 (40–60)	45 (40–60)	40 (40–60)	40 (40–60)
Administration of IVGC, *n* (%)	15 (9.4)	2 (5.1)	3 (15)	1 (2.7)	21 (8.2)
Successful GC discontinuation, *n* (%)	45 (28)	12 (31)	6 (30)	7 (18)	70 (27)
Any GC-sparing agent, *n* (%)	32 (20)	25 (64)	12 (60)	3 (7.9)	72 (28)
Methotrexate, *n* (%)	32 (20)	24 (62)	12 (60)	3 (7.9)	71 (28)
Leflunomide, *n* (%)	0	2 (5.1)	0	0	2 (0.8)
Tocilizumab, *n* (%)	2 (1.3)	4 (10)	5 (25)	1 (2.6)	12 (4.7)
Relapse					
At least one relapse, *n* (%)	17 (11)	15 (38)	9 (45)	11 (29)	52 (20)
Number of relapses, *n* (%)					
1	14 (8.8)	11 (28)	4 (20)	10 (26)	39 (15)
2	2 (1.3)	3 (7.7)	3 (15)	1 (2.6)	9 (3.5)
>2	1 (0.6)	1 (2.6)	2 (10)	0	4 (1.6)
First or second relapse classified as major relapse, *n* (%)	2 (1.3)	3 (7.7)	6 (30)	2 (5.3)	13 (5.1)

Diagnostic delay: time from first symptom to diagnosis; GC: glucocorticoids; IVGC: intravenous glucocorticoids; IQR: interquartile range.

a Presented as ‘median (IQR)’.

To assess predictors of initial GC dosage and the use of intravenous GC (IVGC), we used linear regression and logistic regression, respectively. For linear regressions, we present the coefficients with 95% CI and *P*-value. For logistic regressions, we present the exponentiated coefficients as odds ratios (OR) with 95% CI and *P*-value (Table 2).

**Table 2.** Predictors for initial oral GC dosage and the use of intravenous GC

	*N* (%)^a^	Initial oral GC dosage		IVGC	
Patient characteristic	*N* = 255	β (95% CI)	*P*-value	OR (95% CI)	*P*-value
Age at diagnosis (years)		−0.06 (−0.26 to 0.14)	0.55	0.96 (0.89 to 1.03)	0.29
Male sex	76 (30%)	−1.6 (−5.3 to 2.2)	0.41	1.19 (0.33 to 4.00)	0.78
Time from first symptom to diagnosis (days)		−0.01 (−0.03 to 0.01)	0.36	1.00 (1.00 to 1.01)	0.13
Administration of IVGC	21 (8.3%)	3.3 (−2.9 to 9.4)	0.3		
Localized headache	200 (78%)			0.27 (0.07 to 0.99)	0.049
Scalp tenderness	101 (40%)	−4.4 (−7.8 to −0.98)	0.012		
Polymyalgia rheumatica^b^	72 (28%)				
Morning stiffness in the shoulders or neck^b^	75 (29%)				
Constitutional symptoms	176 (69%)			0.27 (0.07 to 0.95)	0.041
Jaw claudication^b^	121 (47%)				
Limb claudication^b^	11 (4.3%)				
Tenderness over temporal artery^b^	117 (46%)				
Tenderness over other artery^b^	1 (0.4%)				
Reduced pulse in temporal artery	67 (26%)	2.5 (−1.3 to 6.3)	0.19	2.77 (0.75 to 10.2)	0.12
Reduced pulse in other site^b^	8 (3.1%)				
Vascular bruit^b^	9 (3.5%)				
Positive temporal artery biopsy	166 (71%)	4.9 (0.82 to 9.0)	0.019	4.58 (0.85 to 40.4)	0.079
Large vessel involvement	58 (23%)	4.0 (−1.1 to 9.1)	0.13	3.39 (0.45 to 25.0)	0.23
Visual disturbances, including blindness	77 (30%)	12 (7.9 to 16)	<0.001	18.6 (4.81 to 103)	<0.001
ESR (mm/h)		0.03 (−0.04 to 0.10)	0.35	1.00 (0.97 to 1.03)	0.88
CRP (mg/L)		−0.02 (−0.05 to 0.01)	0.25	1.01 (1.00 to 1.02)	0.042
Platelet count (×10^9^/L)				0.99 (0.99 to 1.00)	0.03

Final multivariable predictor models for initial oral GC dosage and IVGC. For initial oral GC dosage, the β can be interpreted as difference in mean GC dosage (mg/day) compared to the reference. For IVGC OR can be interpreted as odds of receiving IVGC compared to the reference. For sex, female sex is the reference, and for all other characteristics, the absence of the given characteristic is the reference. For continuous variables, β and OR can be interpreted as change per unit increase in each variable. β: linear regression coefficient; OR: odds ratio; GC: glucocorticoid; IVGC: intravenous glucocorticoid.

a *N* (%) shown for all categorical variables shows the number (%) of patients with the presence of a given characteristic. For continuous variables, this is empty.

b Variables included in the uni- and full multivariate model, but not included in the final models.

For variable selection in the predictor analyses, we used univariable and multivariable regression models and included variables in a final multivariable regression model only if their *P*-value was less than 0.1 in the univariable or full multivariable model, or if they were of particular clinical interest (age at diagnosis, sex, CRP, ESR, TAB, and LV-GCA). As we wanted to evaluate the associations between treatment decisions and the underlying traits defining each phenotype, we chose to include these underlying traits, that is, TAB and LV-GCA, instead of the phenotypes themselves in the main analysis. Models with phenotypes as predictors are available as Supplementary Material, available at *Rheumatology Advances in Practice* online.

**Figure 2.**
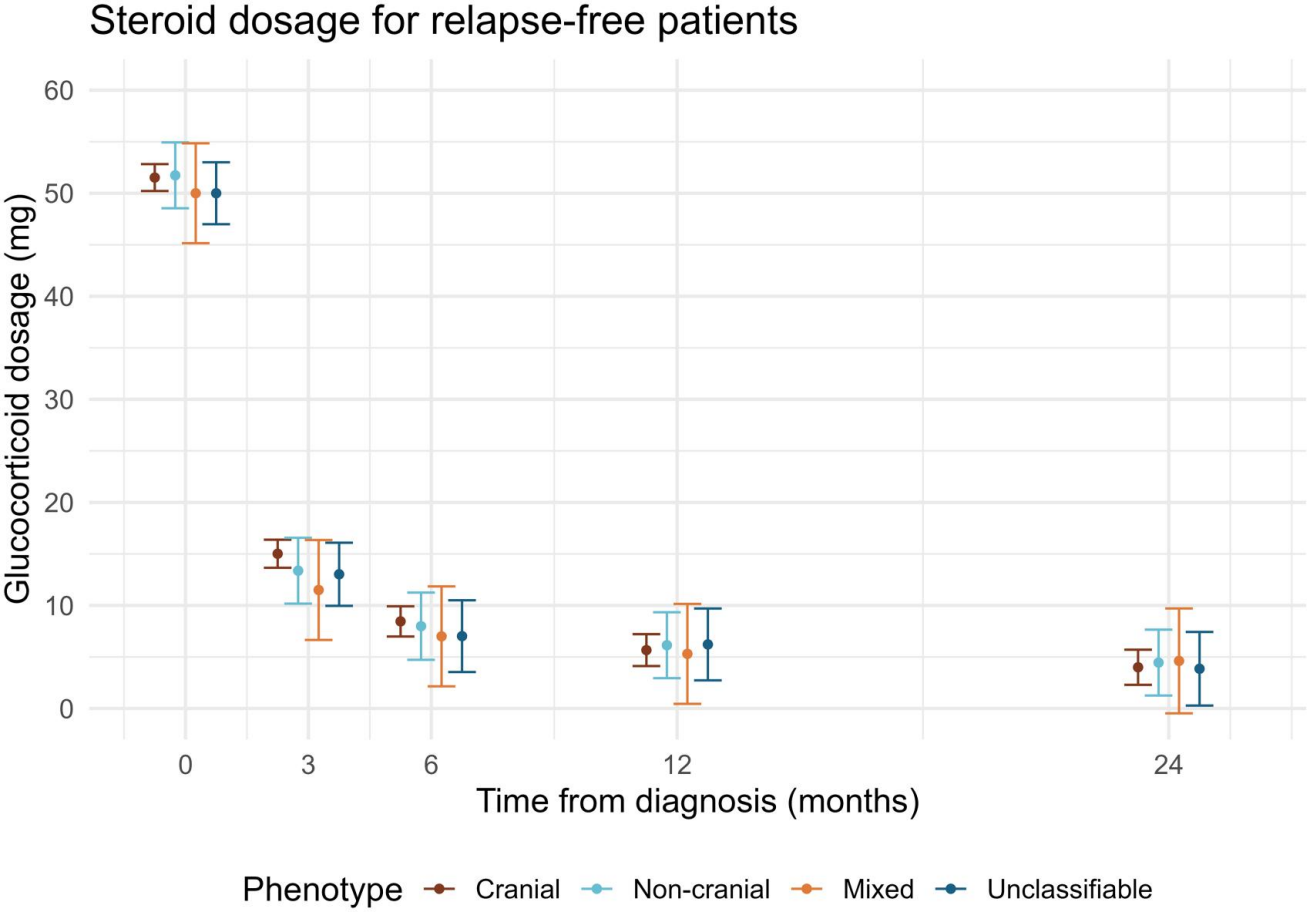
Glucocorticoid tapering by phenotype. Estimated mean glucocorticoid dosage at treatment initiation and at 3, 6, 12, and 24 months of follow-up for each GCA phenotype. Estimates are based on a linear mixed effects model with error bars indicating 95% CIs

The tapering of GC dosage was assessed by a linear mixed effects (LME) model with the interaction between time and phenotype as the main independent fixed effect, GC dosage at the different time points as dependent variable, and adjusted for individual random intercept. As the trend deviated heavily from a linear curve, we used categorical time in the model. We explored models allowing for random slope for each patient, and with age, sex, and a combination of clinical variables as adjustment variables. We evaluated the models by estimating the mean dosage for each phenotype at each time point with corresponding CI (Fig. 2 and Supplementary Fig. S1, available at *Rheumatology Advances in Practice* online).

**Figure 3.**
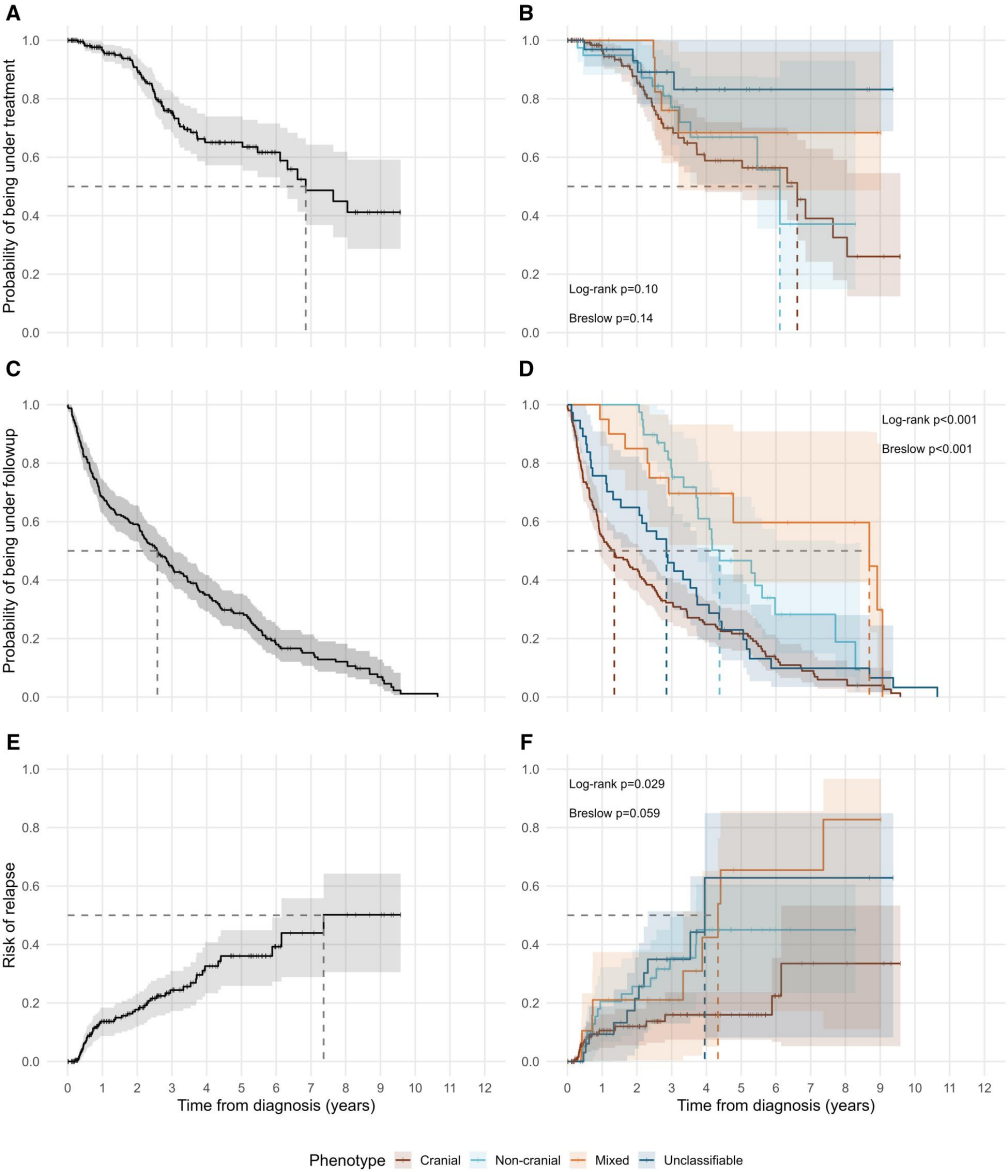
Glucocorticoid discontinuation, rheumatological follow-up, and relapse risk. Kaplan–Meier plots (lines) with CIs (shaded areas) for the complete cohort and by phenotype, respectively, showing glucocorticoid discontinuation (A, B), rheumatological follow-up (C, D), and risk of relapse (E, F). Dashed lines represent median time. *P*-values show the results of log-rank and Gehan–Breslow–Wilcoxon test for the corresponding plot

Follow-up time, time to GC discontinuation, and time to relapse were analysed using time-to-event methods. Kaplan–Meier plots were made for all three outcomes, and log-rank and Gehan–Breslow–Wilcoxon tests were used to evaluate differences between phenotypes (Fig. 3A–F). Individuals were censored upon end of follow-up by the rheumatology department, death, or at the end of the study period. In the time-to-event analysis of GC discontinuation, 12 patients with documented successful discontinuation had to be excluded due to unknown time of discontinuation. We applied Cox regression for multivariable analysis and evaluation of continuous variables. For all three outcomes, we included age at diagnosis, sex, phenotype, initial oral GC dosage and the use of IVGC as variables in a univariable and multivariable model (Supplementary Tables S1–S3, available at *Rheumatology Advances in Practice* online). In the final models, we included age at diagnosis and sex as possible confounders, while GC starting dosage and IVGC were included if their *P*-values were less than 0.1 in the univariable or full multivariable models (Table 3). Results of Cox regression models are reported as hazard ratios (HR) with corresponding 95% CI and *P*-values. Global *P*-values for variables with more than two categories are estimated using a built-in ANOVA method in the R-package gtsummary [25]. All analyses and generation of figures and tables were performed with the statistical software R v. 4.4.1 [26, 27].

**Table 3.** Steroid discontinuation, follow-up, and relapse according to selected variables

	Steroid discontinuation		Follow-up		Relapse	
Patient characteristic	HR (95% CI)	*P*-value	HR (95% CI)	*P*-value	HR (95% CI)	*P*-value
Age at diagnosis	0.99 (0.95 to 1.02)	0.49	1.03 (1.01 to 1.05)	0.002	1.01 (0.98 to 1.05)	0.47
Male sex	1.03 (0.59 to 1.79)	0.92	0.90 (0.66 to 1.22)	0.5	1.39 (0.78 to 2.49)	0.27
Phenotype		0.04		<0.001		0.02
Cranial	—		—		—	
Non-cranial	0.71 (0.35 to 1.46)		0.48 (0.30 to 0.76)		2.68 (1.24 to 5.79)	
Mixed	0.53 (0.21 to 1.36)		0.33 (0.17 to 0.63)		2.83 (1.24 to 6.48)	
Unclassifiable	0.28 (0.10 to 0.80)		0.82 (0.56 to 1.22)		2.59 (1.14 to 5.89)	
Initial oral glucocorticoid dose	0.98 (0.96 to 1.00)	0.043				
Administration of intravenous glucocorticoids					1.87 (0.82 to 4.24)	0.16

Associations between three outcomes and selected variables. For all three outcomes, HR from the final multivariable regression models are shown. For steroid discontinuation, HR can be interpreted as the probability of discontinuing GC treatment compared with a reference group for categorical variables and change per unit increase for continuous variables. For follow-up, HR can be interpreted as the probability of having follow-up ended, and for relapse, HR can be interpreted as the probability of relapse. HR = hazard ratios.

The study was approved by the Norwegian Regional Committees for Medical and Health Research Ethics (REK) (reference number 264780), who waived the need for prior patient consent in accordance with Norwegian law.

## Results

### Population characteristics

The mean age at diagnosis was 72.2 (S.D. = 8.9) years, with 70% (*n* = 180) female patients. Table 1 shows the population characteristics in detail. Among the 72 patients (28.1%) who got steroid-sparing DMARDs, six patients used methotrexate for other diseases before they got their GCA diagnosis. The proportion of patients who received a steroid-sparing agent differed between the phenotypes and was higher for patients with LV-GCA compared with the other phenotypes (*P* < 0.001, Table 1).

### Predicting factors for initial GC treatment

Data on the initial oral GC dosage were available for 255 (99.6%) patients. Visual disturbances and a positive TAB were associated with mean initial dosages of 12.0 mg (*P* < 0.001) and 4.9 mg (*P* = 0.019), respectively, higher than for patients without these traits (Table 2). Scalp tenderness was the only trait associated with a lower mean initial dosage (*P* = 0.012, Table 2).

Visual disturbances (*P* < 0.001) and CRP (*P* = 0.042) were positively associated with the administration of IVGC, while patients with localized headache (*P* = 0.049) and constitutional symptoms (*P* = 0.041) were less likely to receive IVGC (Table 2). The complete uni- and multivariable regression models can be found in Supplementary Tables S4 and S5, available at *Rheumatology Advances in Practice* online. Analyses with phenotype as a predictor variable instead of positive TAB and LV-GCA showed comparable results (Supplementary Tables S6 and S7, available at *Rheumatology Advances in Practice* online).

### GC tapering and discontinuation

Among non-relapsing patients (*n* = 198), GC dosages were available for 99.5% of patients at treatment initiation (*n* = 197), 92.9% at 3 months (*n* = 184), 80.3% at 6 months (*n* = 159), 74.7% at 12 months (*n* = 148), and 64.6% at 24 months (*n* = 128). The GC dosage for non-relapsing patients at each time point did not differ between the phenotypes (Fig. 2). We also examined the effect of allowing a random slope for each individual, but it did not yield a different result, nor did the inclusion of possible confounders (age, sex, and visual symptoms). Patients with visual symptoms received higher mean initial dosage compared with patients without visual symptoms, but dosages were not significantly different at 3, 6, 12, and 24 months (Supplementary Fig. S1, available at *Rheumatology Advances in Practice* online).

Seventy (27.3%) patients successfully discontinued GC therapy. The median time to GC discontinuation was 6.85 years (Fig. 3A). Patients with cranial phenotype were more likely to discontinue GC during follow-up than the other phenotypes (*P* = 0.04, Table 3), while those with a higher initial GC dosage were less likely to discontinue (*P* = 0.043, Table 3).

### Follow-up time and relapse

The median follow-up time at the rheumatological care facility was 2.58 years (Fig. 3C). Patients with the cranial phenotype had shorter follow-up time than the other subgroups (*P* < 0.001, Fig. 3D). Furthermore, higher age at time of diagnosis was associated with shorter period of follow-up, whereas the duration of follow-up was unaffected by sex (Table 3).

Fifty-two (20.3%) patients experienced relapse during their follow-up. The median time to relapse was 11.2 months (95% CI 8.3–26.4 months), indicating that most of the relapsing patients experienced their first relapse during the first 2 years after diagnosis. Patients with the cranial phenotype were less likely to relapse than patients with the other phenotypes (*P* = 0.029, Fig. 3F). This pattern persisted after adjustment for age, sex, and the administration of IVGC (Table 3). The complete uni- and multivariable regression models for GC discontinuation, follow-up time and relapse can be found in Supplementary Tables S1–S3, available at *Rheumatology Advances in Practice* online.

## Discussion

In this study, we found that individuals with the cranial GCA phenotype have a lower risk of relapse compared with patients with non-cranial, mixed, and unclassifiable phenotypes. Patients with cranial phenotype also tended to be followed for a shorter period of time at the rheumatological care facility. However, GC treatment was not dependent on phenotype, neither regarding initial dosage nor tapering. The factor most strongly associated with a higher mean oral GC dosage and administration of IVGC was visual disturbances.

Previous studies that have assessed the frequency of relapse in GCA patients have shown diverging results. A recent, large meta-analysis found that in observational and population-based studies, the proportion of GCA patients experiencing relapse ranged from 20% to 79% in different studies [15]. The findings from our study fall in the lower range of this interval. Female sex has been associated with higher relapse risk in some studies [23, 28, 29], but not in others [24, 30, 31]. The most consistently shown risk factor for a relapsing disease course is LV-GCA, which has repeatedly been associated with relapse [17, 19, 23, 24, 29]. In a recent meta-analysis, Moreel *et al*. found three factors to be associated with increased relapse risk, namely LV-GCA, female sex, and lower age [32]. The analyses in this article do not constitute a full prediction model for relapse, but rather a targeted analysis of the association between phenotype and relapse risk, adjusted for age, sex, initial GC dosage, and IVGC. Our main finding was the association between phenotype and relapse risk. We, however, did not find an association with age and sex. Interestingly, none of the studies included in Moreel’s meta-analysis originated from Nordic countries.

Moreel *et al*. evaluated the timing of relapse in a meta-analysis, and found that the risk was highest in the first 2 years after diagnosis [32]. This is concordant with our study, although after the first year, we see a different progression of the occurrence of relapses for the cranial phenotype compared with the other phenotypes.

Our findings of shorter follow-up time, higher probability of GC discontinuation, and lower risk of relapse for patients with cranial phenotype could suggest that they tend to have a less complicated disease course compared with patients with the other phenotypes. This has, to the best of our knowledge, not been shown in previous studies. It is important to recognize that age is an important factor in any healthcare setting, and that age is not independent of phenotype [3]. In our analysis of follow-up time, we expected and confirmed age at diagnosis to be a confounder, that is, being associated both with the outcome (ending of rheumatological follow-up) and the exposure (phenotype). Still, after adjustment for age, phenotype remained significantly associated with all the outcomes in our analyses.

The use of IVGC was low, compared with a recent Swedish population-based study [33]. The patients in our study were generally treated according to Norwegian and European recommendations [4, 16]. At present, there are no guidelines indicating conclusively that GCA treatment should be directed by phenotype. Previous EULAR recommendations advocated the administration of steroid-sparing treatment to all patients with LV-GCA, but in the most recent update, this recommendation is moderated to comprise selected patients with refractory or relapsing disease or at risk of GC-related complications [16]. In our study, a larger proportion of patients with non-cranial and mixed phenotypes received a steroid-sparing agent during the disease course compared with patients with cranial and unclassifiable phenotypes. A likely explanation is difficulties in tapering the GCs. As this is an observational study, the treating physicians were not blinded regarding disease phenotype. However, it is assumed that treatment decisions were primarily guided by medical indications. This association between phenotype and the use of adjunctive therapy has, to the best of our knowledge, not been shown previously in a real-world cohort of consecutive GCA patients.

Almost all the patients who received a steroid-sparing agent in our study received methotrexate, while a few were treated with tocilizumab. de Mornac *et al*. found a similar trend in their cohort, although percentages are not directly comparable as their study included only patients examined with large vessel imaging [23]. The Norwegian pharmaceutical tender system did not include tocilizumab for GCA prior to October 2022 [34]. Thus, during most of the study and follow-up period, tocilizumab was not widely available. This is probably the main reason for the low number of patients treated with tocilizumab.

GC discontinuation was documented in only 27.3% of the patients, with a median time from diagnosis to discontinuation of 6.85 years. Current guidelines provide recommendations for the minimal treatment duration for GCA patients [4, 16]. Still, the optimal time point for GC discontinuation is not well defined, and GC treatment is commonly continued for several years, resulting in high cumulative GC doses [35]. This is despite the fact that long-term low-dosage GC has been associated with adverse effects such as osteoporosis and arterial hypertension [36].

A limitation of our study was the small number of patients in some of the phenotype groups studied. Although the number of included patients in the complete cohort is comparatively large, sufficient statistical power may be difficult to achieve in some subgroup analyses. This contributes to the uncertainty in our estimates and increases the risk of a Type II error, that is, failure to detect a significant difference. This should be considered when the results are interpreted, especially results that are diverging from other studies. Other limitations of our study include the retrospective data collection and the inherent assumptions required by the statistical models, particularly concerning uncertainties regarding missingness in the longitudinal data.

A strength of our study is the completeness and quality of the study cohort, as well as the relatively high number of patients on long follow-up time. Nonetheless, we do not know what happened to the patients after the follow-up at the rheumatology care facility ended, which constitutes a limitation regarding possible late relapses. Still, our follow-up period is comparable to other studies [23, 37]. In earlier studies, different patient selections have been used, and some studies included only patients with certain characteristics, such as selection of only TAB-positive patients [38], only patients having undergone large vessel examination [23], or only patients with a minimum follow-up time [29, 39]. All these selection methods could possibly introduce a selection bias. Also, the fact that the definitions of relapse applied in the various studies are differing complicates comparison of studies. Nonetheless, our study provides findings not previously described in a cohort from the Nordic countries, a population known for having one of the highest incidences of GCA.

## Conclusions

In our study, fewer GCA patients with the cranial phenotype experienced relapse compared with the other phenotypes. Patients with the cranial phenotype also tended to have shorter follow-up in the rheumatology department and were more likely to succeed in GC discontinuation. The initial GC treatment of GCA patients did not differ between the phenotypes, and the only factor strongly associated with more intensive initial treatment was visual disturbances. Altogether, our study could suggest that early phenotype classification can yield important prognostic information. Further research is needed to fully understand how this knowledge can be implemented in clinical practice, and to determine whether patients with different phenotypes should be managed differently from the initiation of clinical care.

## Supplementary Material

rkaf109_Supplementary_Data

## Data Availability

The data from this article cannot be made publicly available due to data protection regulations. Aggregated data can be considered shared upon request to the corresponding author.
