# Supplementary material for: Treatment and risk of relapse in GCA in Western Norway 2013–2020: a retrospective cohort study
Source: Rheumatol Adv Pract. 2025 Sep 20;9(4):rkaf109. doi: 10.1093/rap/rkaf109 (PMC12560782; doi:10.1093/rap/rkaf109)
Supplement: rkaf109_Supplementary_Data [file rkaf109_supplementary_data.zip › 25-061_Supplementary_Figure_S1.docx]

**
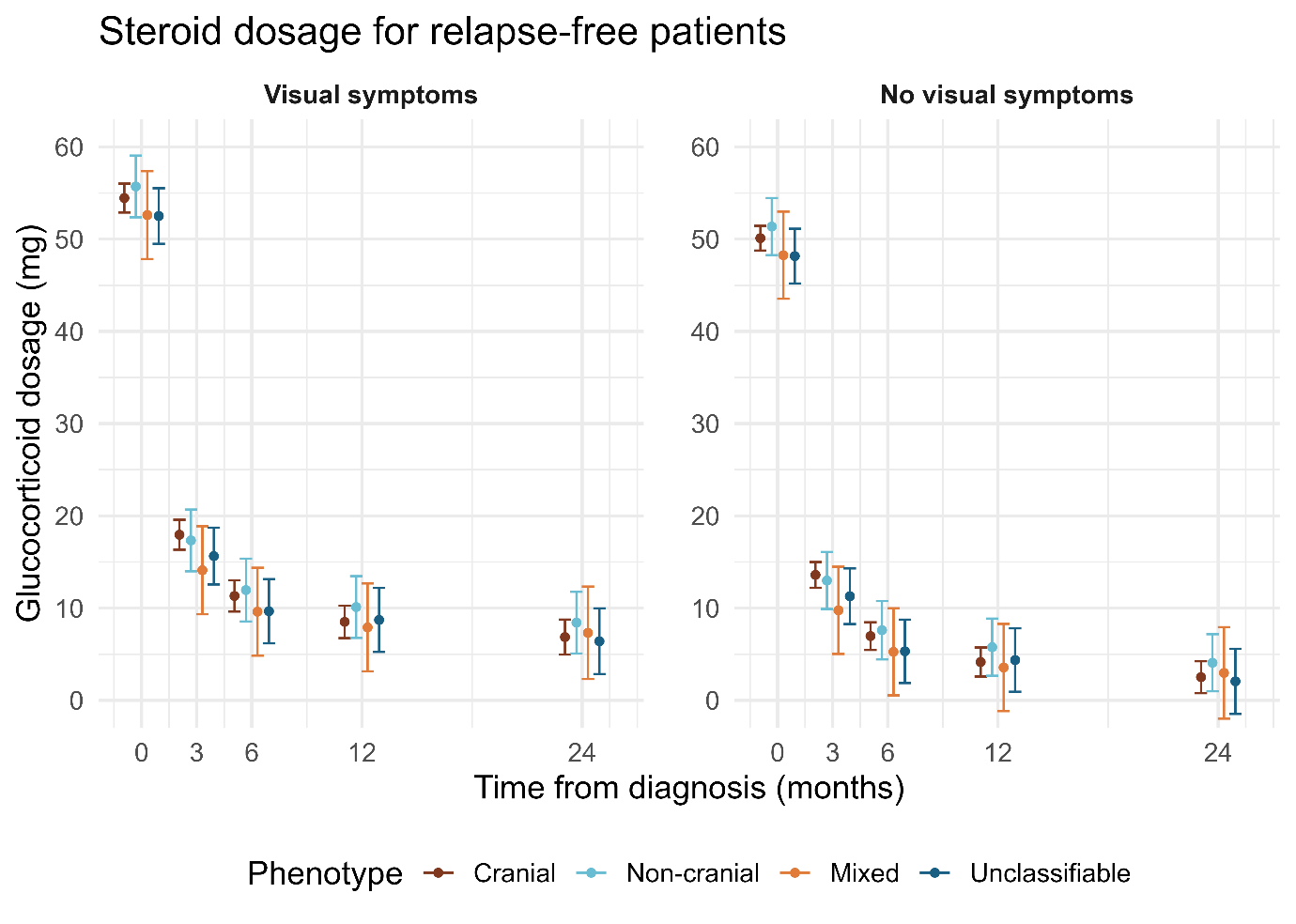
**

**Supplementary Figure S1 – Glucocorticoid tapering by phenotype and visual symptoms**

Estimated mean steroid dosage at treatment initiation, and at 3, 6, 12, and 24 months of follow-up for each GCA-phenotype shown for patients with and without visual symptoms. Estimates are based on a linear mixed effects model with error bars indicating 95% confidence intervals.

**Alt text:** Graph depicting the estimated mean glucocorticoid dosage by phenotype and time for patients with and without visual disturbances.
